# Supplementary material for: Association of pneumococcal carriage in infants with the risk of carriage among their contacts in Nha Trang, Vietnam: A nested cross-sectional survey
Source: PLoS Med. 2022 May 31;19(5):e1004016. doi: 10.1371/journal.pmed.1004016 (PMC9197035; doi:10.1371/journal.pmed.1004016)
Supplement: S1 STROBE Checklist — STROBE, Strengthening the Reporting of Observational Studies in Epidemiology. (PDF) [file pmed.1004016.s001.pdf]

STROBE Statement—Checklist of items that should be included in reports of ***cross-sectional studies***

	Item No	Recommendation	Page No
**Title and abstract**	1	(*a*) Indicate the study's design with a commonly used term in the title or the abstract	Title and Paragraph 2 in Abstract
		(*b*) Provide in the abstract an informative and balanced summary of what was done and what was found	Abstract
**Introduction**			
Background/rationale	2	Explain the scientific background and rationale for the investigation being reported	Paragraph 1,2,3 in Introduction
Objectives	3	State specific objectives, including any prespecified hypotheses	Paragraph 4 in Introduction
**Methods**			
Study design	4	Present key elements of study design early in the paper	Paragraph 3 in Methods
Setting	5	Describe the setting, locations, and relevant dates, including periods of recruitment, exposure, follow-up, and data collection	Paragraph 1,3,4,5 in Methods
Participants	6	(*a*) Give the eligibility criteria, and the sources and methods of selection of participants	Paragraph 2,3 in Methods
Variables	7	Clearly define all outcomes, exposures, predictors, potential confounders, and effect modifiers. Give diagnostic criteria, if applicable	Paragraph 4,5,9,10,11 in Methods
Data sources/ measurement	8*	For each variable of interest, give sources of data and details of methods of assessment (measurement). Describe comparability of assessment methods if there is more than one group	Paragraph 4,5,9,11 in Methods
Bias	9	Describe any efforts to address potential sources of bias	Paragraph 2 in Methods
Study size	10	Explain how the study size was arrived at	Paragraph 2,3 in Methods
Quantitative variables	11	Explain how quantitative variables were handled in the analyses. If applicable, describe which groupings were chosen and why	Paragraph 7,8 in Methods, 3,4 in Results, Table 1, SI Table 3
Statistical methods	12	(*a*) Describe all statistical methods, including those used to control for confounding	Paragraph 7,8,9,10,11,12 in Methods
		(*b*) Describe any methods used to examine subgroups and interactions	Not applicable
		(*c*) Explain how missing data were addressed	Paragraph 1 in Results
		(*d*) If applicable, describe analytical methods taking account of sampling strategy	Not applicable
		(*e*) Describe any sensitivity analyses	Not applicable
**Results**			

Participants	13*	(a) Report numbers of individuals at each stage of study—eg numbers potentially eligible, examined for eligibility, confirmed eligible, included in the study, completing follow-up, and analysed	Paragraph 1 in Results
		(b) Give reasons for non-participation at each stage	Paragraph 1 in Results
		(c) Consider use of a flow diagram	Considered and did not use.
Descriptive data	14*	(a) Give characteristics of study participants (eg demographic, clinical, social) and information on exposures and potential confounders	Paragraph 1 in Results and Table 1
		(b) Indicate number of participants with missing data for each variable of interest	Paragraph 1 in Results
Outcome data	15*	Report numbers of outcome events or summary measures	Paragraph 2,3,4 in Results
Main results	16	(*a*) Give unadjusted estimates and, if applicable, confounder-adjusted estimates and their precision (eg, 95% confidence interval). Make clear which confounders were adjusted for and why they were included	Paragraph 7,8 in Methods, 3,4 in Results, Table 1, SI Table 3.
		(*b*) Report category boundaries when continuous variables were categorized	Paragraph 7,8 in Methods, 3,4 in Results, Table 1, SI Table 3
		(*c*) If relevant, consider translating estimates of relative risk into absolute risk for a meaningful time period	Not relevant
Other analyses	17	Report other analyses done—eg analyses of subgroups and interactions, and sensitivity analyses	Not applicable
**Discussion**			
Key results	18	Summarise key results with reference to study objectives	Paragraph 1 in Discussion
Limitations	19	Discuss limitations of the study, taking into account sources of potential bias or imprecision. Discuss both direction and magnitude of any potential bias	Paragraph 6 in Discussion
Interpretation	20	Give a cautious overall interpretation of results considering objectives, limitations, multiplicity of analyses, results from similar studies, and other relevant evidence	Paragraph 1,2,3,4,5,6,7 in Discussion
Generalisability	21	Discuss the generalisability (external validity) of the study results	Paragraph 1,2,3,4,5 in Discussion
**Other information**			
Funding	22	Give the source of funding and the role of the funders for the present study and, if applicable, for the original study on which the present article is based	Funding

*Give information separately for exposed and unexposed groups.

Note: An Explanation and Elaboration article discusses each checklist item and gives methodological background and published examples of transparent reporting. The STROBE checklist is best used in conjunction with this article (freely available on the Web sites of PLoS Medicine at http://www.plosmedicine.org/, Annals of Internal Medicine at http://www.annals.org/, and Epidemiology at http://www.epidem.com/). Information on the STROBE Initiative is available at www.strobe-statement.org.
