# Supplementary material for: Association of pneumococcal carriage in infants with the risk of carriage among their contacts in Nha Trang, Vietnam: A nested cross-sectional survey
Source: PLoS Med. 2022 May 31;19(5):e1004016. doi: 10.1371/journal.pmed.1004016 (PMC9197035; doi:10.1371/journal.pmed.1004016)
Supplement: S1 File — (PDF) [file pmed.1004016.s002.pdf]

Bộ câu hỏi phỏng vấn   Mã số _ _ _ _ _ _ _ _

Ngày phỏng vấn: _ _ / _ _ / _ _ _ _

Date of interview:

Xã/phường: ___________________________________

Commune:

Ngày sinh của trẻ : _ _ / _ _ / _ _ _ _
Date of birth of the infant:
Khoảng cách gần nhất từ nhà tới ranh giới địa phận xã/phường là: _______ mét
Approximate distance of household to commune border: (meters)

Mối quan hệ giữa người được phỏng vấn với trẻ: Relationship between the interviewee and the infant: ☐ Mẹ/Mother ☐ Cha/Father
☐ Cô/dì Aunt ☐ Ông/bà Grandparent ☐ Người trông trẻ/Quản gia Baby sister/butler ☐ Khác Other: _________

I. Câu hỏi chung: General information:

1.1 Giới của trẻ: Gender ☐ **Nam/Male** ☐ **Nữ/Female**

1.2 Số anh/chị/em ruột của con anh/chị sống trong cùng một hộ là bao nhiêu cháu? Number of siblings living in the same house?

☐ 0 ☐ 1 ☐ 2 ☐ 3 ☐ >=4

1.3 Tổng số người sống trong hộ, bao gồm trẻ? Number of peoplr in the household, including the infant?

☐ 2 ☐ 3 ☐ 4 ☐ 5 ☐ >=6

1.4 Người chăm sóc trẻ hiện tại có đi làm không? Caretaker currently in a paid employment?

☐ Không đi làm/No ☐ Có, < 2 ngày/tuần/ Yes, less than 2 days a week ☐ Có, ≥ 2 ngày/tuần Yes, at least 2 days a week

1.5 Trình độ học vấn cao nhất trong hộ? Highest level of education in the household?

☐ Mù chữ/None ☐ Tiểu học/Primary school ☐ THCS/Secondary school ☐ Từ THPT trở lên / From high school

1.6 Khả năng vận động hiện tại của trẻ? Infant mobility?

☐ Không/None ☐ Ngồi/ Siting ☐ Bò/ Crawing ☐ Đi/ Walking

II. Những câu hỏi về đi lại Mobility questions (Hỏi trực tiếp câu hỏi này với người chăm sóc chính hoặc người sẽ giám sát trẻ trong ngày thu thập dữ liệu.) (Ask dicrectly the main caretaker or main observer)

2.1. Bây giờ nếu cần phải di chuyển, loại phương tiện nào anh/chị sẽ sử dụng If having a travel now, what means of transport available (đánh dấu tất cả những phương tiện nếu phù hợp)? (tick all that apply)

☐ Xe đạp/bike ☐ Xe máy/mortorbike ☐ Xe Ô tô/car ☐ Đi bộ/walking

☐ Phương tiện công cộng (xe buýt, tàu hỏa) Public transport (bus, train …) ☐ Khác Other ________________

2.2. Trong vòng 07 ngày qua, anh/chị đã rời khỏi xã/phường của mình bao nhiêu lần? Number of times in interviewee left the commune in the last 7 days?

☐ 0 lần time ☐ 1-2 lần times ☐ 3-6 lần times ☐ 7-14 lần times
☐ > 14 lần times

2.3. Trong vòng 7 ngày qua, trẻ đã rời khỏi xã/phường bao nhiêu lần? (Bạn có thể hỏi nhiều lần các thành viên trong gia đình, bao gồm bố/mẹ và ông/bà, để biết được tổng số lần trẻ rời khỏi xã/phường) Number of time the infant left the commune in the last 7 days? (The interviewer can ask the other member in the family, including parent and grandparent to know the total number of times the infant left the commune)

☐ 0 lần times ☐ 1-2 lần times ☐ 3-6 lần times ☐ 7-14 lần times
☐ > 14 lần times

III. Những câu hỏi về chăm sóc trẻ: Childcare question:

3.1 Trẻ có đang đi học ở trường mẫu giáo hoặc nhè trẻ không? Infant attend childcare?

☐ Không/No ☐ MG công lập/Public nursery ☐ MG tư thục/Private nursery
☐ Nhóm trẻ gia đình/ Child minder

Nếu trẻ có đi học: If attending:

3.2. Nơi trẻ học có ở trong xã/phường trẻ đang sống không? Is childcare in the same commune?

☐ Có/Yes ☐ Không/No

3.3 Cháu đến nơi học bao nhiêu ngày trong tuần? How many days a week does infant attend?

☐ 1-2 ngày days ☐ 3-4 ngày days ☐ > 4 ngày days
