# Supplementary material for: Association of pneumococcal carriage in infants with the risk of carriage among their contacts in Nha Trang, Vietnam: A nested cross-sectional survey
Source: PLoS Med. 2022 May 31;19(5):e1004016. doi: 10.1371/journal.pmed.1004016 (PMC9197035; doi:10.1371/journal.pmed.1004016)
Supplement: S3 File — (PDF) [file pmed.1004016.s004.pdf]

Personal communication permission form

PLOS Medicine manuscript ID: PMEDICINE-D-21-02876R1

Title: Pneumococcal exposure routes for infants, a nested cross-sectional survey in Nha Trang, Vietnam

Authors: George Qian, Michiko Toizumi, Sam Clifford, Lien Thuy Le, Tasos Papastylianou, Catherine Satzke, Billy Quilty, Chihiro Iwasaki, Noriko Kitamura, Mizuki Takegata, Minh Xuan Bui, Hien Anh Thi Nguyen, Duc Anh Dang, Albert Jan van Hoek, Lay Myint Yoshida, Stefan Flasche

I, the undersigned, agree to be identified by the authors of the above-named manuscript as the source of a personal communication that provided information of under-five population of Nha Trang in 2018 on October 2nd, 2021.

Name: Dr. Minh Xuan Bui

Affiliation: Khanh Hoa Health Service

Position: Director

Date: Feb 28th, 2022

Signature:
