# Supplementary material for: Association of pneumococcal carriage in infants with the risk of carriage among their contacts in Nha Trang, Vietnam: A nested cross-sectional survey
Source: PLoS Med. 2022 May 31;19(5):e1004016. doi: 10.1371/journal.pmed.1004016 (PMC9197035; doi:10.1371/journal.pmed.1004016)

# Supplemental material to:

# Association of pneumococcal carriage in Vietnamese infants with the risk of carriage among their contacts: a nested cross-sectional survey

Authors: George Qian^1*^, Michiko Toizumi^2*^, Sam Clifford^1^, Lien Thuy Le^3^, Tasos Papastylianou^4^ , Catherine Satzke^5^, Billy Quilty^1^, Chihiro Iwasaki^2^, Noriko Kitamura^2^, Mizuki Takegata^2^, Minh Xuan Bui^6^, Hien Anh Thi Nguyen^7^, Duc Anh Dang^7^, Albert Jan van Hoek^8^, Lay Myint Yoshida^2☨^, Stefan Flasche^1☨^

Institutions:
^1^ Centre for Mathematical Modelling of Infectious Diseases, London School of Hygiene and Tropical Medicine, London, UK
^2^ Institute of Tropical Medicine, Nagasaki University, Nagasaki, Japan
^3^ Department of Bacteriology, the Pasteur Institute in Nha Trang, Nha Trang, Vietnam
^4^ School of Computer Science and Electronic Engineering, University of Essex, Colchester, UK
^5^ Translational Microbiology Group, Murdoch Children's Research Institute at the Royal Children's Hospital, University of Melbourne, Parkville, Australia
^6^ Khanh Hoa Health Service, Nha Trang, Vietnam
^7^ National Institute of Hygiene and Epidemiology, Hanoi, Vietnam
^8^ Centre for Infectious Disease Control, National Institute for Public Health and the Environment, Bilthoven, The Netherlands

## *Model Comparison*

We compared the model in Equation 2 with other logistic regression models with different covariants:

- Carriage ~ Number of Contacts (traditionally the covariate used for predicting carriage)
- Carriage ~ PEI (to justify the inclusion of the other covariates)
- Carriage ~ PEI + Infants’ Age
- Carriage ~ PEI + Commune
- Carriage ~ [(PEI + Infants’ Age) with commune as groups]|longer duration contacts only (including only long-duration contacts)

These models were compared using the Deviance Information Criterion (DIC) values, a generalisation of the Akaike Information Criterion that is suitable for comparison of Bayesian models, provided that the covariates approximately follow a Gaussian distribution; we found that this was indeed the case for the covariates in our model.

The results are shown in Table 5. The model with PEI as the lone covariate appears to outperform the corresponding model with the number of contacts alone, based on DIC values. The set of models with PEI together with covariates such as mobility and motorcycle share similar DIC values and appear to be the best-performing models; this set includes the proposed model (Equation 2). Including only longer-duration contacts does not appear to reduce the DIC value.

**Table A**: Effect of each infant's characteristic on having contact outside of the commune of residence, estimated using logistic regression model.

| Characteristics | | | | | N | % Infants with contacts in other communes | Odds ratio | p-value | Adjusted odds ratio* | p-value | Adjusted odds ratio** |  |
| --- | --- | --- | --- | --- | --- | --- | --- | --- | --- | --- | --- | --- |
| Total | | | | | 1583 | 154 (9.7) |  |  |  |  |  |  |
| Demographics | | | | |  |  |  |  |  |  |  |  |
|  | | Sex | | |  |  |  |  |  |  |  |  |
|  | |  | | Male | 871 | 85 (9.8) | reference | 0.964 | reference | 0.837 |  |  |
|  | |  | | Female | 712 | 69 (9.7) | 0.99 (0.71-1.39) |  | 0.96 (0.66-1.41) |  |  |  |
|  | | Age (months) | | |  |  |  |  |  |  |  |  |
|  | |  | | <8 months | 616 | 44 (7.1) | reference | 0.006 |  |  | reference | 0.024 |
|  | |  | | 8-13 months | 967 | 110 (11.4) | 1.67 (1.16-2.40) |  |  |  | 1.41 (1.05-1.90)*** |  |
| Family | | | | |  |  |  |  |  |  |  |  |
|  | | Siblings in the household | | |  |  |  |  |  |  |  |  |
|  | |  | | No sibling | 666 | 66 (9.9) | reference | 0.835 | reference | 0.738 |  |  |
|  | |  | | One or more siblings | 917 | 88 (9.6) | 0.97 (0.69-1.35) |  | 0.96 (0.74-1.23) |  |  |  |
|  | | Number of people in the household | | |  |  |  |  |  |  |  |  |
|  | |  | | 2-4 | 611 | 62 (10.2) | reference | 0.656 | reference | 0.655 |  |  |
|  | |  | | >4 | 972 | 92 (9.5) | 0.93 (0.66-1.3) |  | 0.91 (0.62-1.36) |  |  |  |
|  | | Caretaker currently in a paid employment | | |  |  |  |  |  |  |  |  |
|  | |  | | No | 1056 | 86 (8.1) | reference | 0.003 | reference | 0.008 | reference | 0.702 |
|  | |  | | Yes | 527 | 68 (12.9) | 1.67 (1.19-2.34) |  | 1.60 (1.13-2.27) |  | 1.08 (0.71-1.65) |  |
|  | | Highest level of education in the household | | |  |  |  |  |  |  |  |  |
|  | |  | | Secondary school or lower | 268 | 27 (10.1) | reference | 0.834 | reference | 0.734 |  |  |
|  | |  | | Degree | 1315 | 127 (9.7) | 0.95 (0.62-1.48) |  | 0.94 (0.65-1.36) |  |  |  |
| Infant's activity | | | | |  |  |  |  |  |  |  |  |
|  | | Sit | | |  |  |  |  |  |  |  |  |
|  | |  | | Yes | 1334 | 139 (10.4) | 1.81 (1.05-3.15) | 0.034 | 1.36 (0.70-2.62) | 0.364 |  |  |
|  | |  | | No | 249 | 15 (6.0) | reference |  | reference |  |  |  |
|  | | Crawl | | |  |  |  |  |  |  |  |  |
|  | |  | | Yes | 873 | 95 (10.9) | 1.35 (0.96-1.9) | 0.087 | 1.08 (0.73-1.59) | 0.713 |  |  |
|  | |  | | No | 710 | 59 (8.3) | reference |  | reference |  |  |  |
|  | | Walk | | |  |  |  |  |  |  |  |  |
|  | |  | | Yes | 186 | 28 (9.0) | 1.79 (1.15-2.78) | 0.01 | 1.51 (0.95-2.39) | 0.079 |  |  |
|  | |  | | No | 1397 | 126 (15.1) | reference |  | reference |  |  |  |
| Mobility | | | | |  |  |  |  |  |  |  |  |
|  | | Bicycle | | |  |  |  |  |  |  |  |  |
|  | |  | | Yes | 31 | 1 (3.2) | 0.30 (0.04-2.25) | 0.244 | 0.30 (0.04-2.32) | 0.248 |  |  |
|  | |  | | No | 1552 | 153 (9.9) | reference |  | reference |  |  |  |
|  | | Motorbike | | |  |  |  |  |  |  |  |  |
|  | |  | | Yes | 1549 | 152 (9.8) | 1.74 (0.41-7.34) | 0.45 | 1.73 (0.30-9.9) | 0.541 |  |  |
|  | |  | | No | 34 | 2 (5.9) | reference |  | reference |  |  |  |
|  | | Car | | |  |  |  |  |  |  |  |  |
|  | |  | | Yes | 99 | 12 (12.1) | 1.3 (0.7-2.44) | 0.408 | 1.34 (0.78-2.31) | 0.283 |  |  |
|  | |  | | No | 1484 | 142 (9.6) | reference |  | reference |  |  |  |
|  | | Walk | | |  |  |  |  |  |  |  |  |
|  | |  | | Yes | 108 | 10 (9.3) | 0.94 (0.48-1.85) | 0.865 | 0.97 (0.50-1.87) | 0.918 |  |  |
|  | |  | | No | 1475 | 144 (9.8) | reference |  | reference |  |  |  |
|  | | Public transportation | | |  |  |  |  |  |  |  |  |
|  | |  | | Yes | 40 | 5 (12.5) | 1.34 (0.52-3.46) | 0.55 | 1.44 (0.68-3.03) | 0.342 |  |  |
|  | |  | | No | 1543 | 149 (9.7) | reference |  | reference |  |  |  |
|  | | Number of times caretaker left the commune in the last 7 days | | |  |  |  |  |  |  |  |  |
|  | |  | | 0-2 | 872 | 44 (5.1) | reference | <0.001 | reference | <0.001 | reference | <0.001 |
|  | |  | | 3 or more | 711 | 110 (15.5) | 3.44 (2.39-4.96) |  | 3.36 (2.02-5.58) |  | 3.01 (1.78-5.10) |  |
|  | | Number of times infant left the commune in the last 7 days | | |  |  |  |  |  |  |  |  |
|  | |  | | 0 | 788 | 9 (1.1) | reference | <0.001 | reference | <0.001 | reference | <0.001 |
|  | |  | | 1 or more | 795 | 145 (18.2) | 19.31 (9.77-38.16) |  | 18.9 (7.99-44.72) |  | 18.3 (7.87-42.47) |  |
| Day-care | | | | |  |  |  |  |  |  |  |  |
|  | | Day-care attendance | | |  |  |  |  |  |  |  |  |
|  | |  | | Yes | 1497 | 121 (8.1) | 7.08 (4.41-11.36) | <0.001 | 6.56 (3.92-10.98) | <0.001 |  |  |
|  | |  | | No | 86 | 33 (38.4) | reference |  | reference |  |  |  |
| Pneumococcus carriage (n=1582) | | | | |  |  |  |  |  |  |  |  |
|  | | Pneumococcus | | |  |  |  |  |  |  |  |  |
|  | |  | | Yes | 353 | 48 (13.6) | 1.67 (1.16-2.40) | 0.006 | 1.58 (1.13-2.21) | 0.008 | 1.25 (0.92-1.71) | 0.159 |
|  | |  | | No | 1229 | 106 (8.6) | reference |  | reference |  | reference |  |
|  | *Odds ratios adjusted by age group, considering clustering in each commune | | | | | | | | |  |  |  |
|  |  | | **Odds ratios adjusted by age group & day-care attendance, considering clustering in each commune | | | | | | | | |  |
|  |  | | ***Odds ratio adjusted by day-care attendance, considering clustering in each commune | | | | | | | | |  |

**Table B:** A comparison of different logistic regression models to predict carriage, using Deviance Information Criterion (DIC) values

| **Covariates** | **DIC value** |
| --- | --- |
| Number of Contacts | 1682 |
| PEI | 1656 |
| PEI + Infants’ Age | 1618 |
| PEI+Infants’ Age, with Commune as a random effect | 1618 |
| PEI + Infants’ Age - Longer Duration Contacts Only, with Commune as a random effect | 1619 |

PEI: Pneumococcal Exposure Index

**Table C:** Values of the coefficients from the logistic regression (Equation 2) are given here.

| **Parameter** | **Mean Value** | **2.5%** | **97.5%** |
| --- | --- | --- | --- |
| Fixed-effects model Intercept ($\beta_{0}$) | -3.33 | -4.82 | -2.05 |
| Effect of PEI ($\beta_{1}$) | 1.96 | 1.40 | 2.51 |
| Commune ID: 21 ($C_{1}$) | 0.13 | -0.39 | 0.64 |
| Commune ID: 17 ($C_{2}$) | 0.21 | -0.32 | 0.71 |
| Commune ID: 16 ($C_{3}$) | 0.06 | -0.46 | 0.57 |
| Commune ID: 23 ($C_{4}$) | 0.01 | -0.52 | 0.53 |
| Commune ID: 18 ($C_{5}$) | -0.11 | -0.68 | 0.43 |
| Commune ID: 14 ($C_{6}$) | 0.18 | -0.32 | 0.70 |
| Commune ID: 27 ($C_{7}$) | -0.12 | -0.66 | 0.41 |
| Commune ID: 10 ($C_{8}$) | -0.16 | -0.71 | 0.37 |
| Commune ID: 7 ($C_{9}$) | 0.05 | -0.50 | 0.58 |
| Commune ID: 3 ($C_{10}$) | -0.43 | -1.09 | 0.14 |
| Commune ID: 25 ($C_{11}$) | -0.40 | -1.03 | 0.17 |
| Commune ID: 20 ($C_{12}$) | 0.39 | -0.13 | 0.90 |
| Commune ID: 13 ($C_{13}$) | 0.11 | -0.43 | 0.61 |
| Commune ID: 11 ($C_{14}$) | -0.02 | -0.58 | 0.52 |
| Commune ID: 5 ($C_{15}$) | -0.16 | -0.73 | 0.35 |
| Commune ID: 15 ($C_{16}$) | 0.55 | 0.05 | 1.05 |
| Commune ID: 24 ($C_{17}$) | 0.32 | -0.20 | 0.84 |
| Commune ID: 12 ($C_{18}$) | -0.66 | -1.34 | -0.07 |
| Commune ID: 1 ($C_{19}$) | -0.15 | -0.71 | 0.38 |
| Commune ID: 4 ($C_{20}$) | 0.01 | -0.51 | 0.50 |
| Commune ID: 19 ($C_{21}$) | -0.37 | -0.96 | 0.17 |
| Commune: ID 22 ($C_{22}$) | 0.09 | -0.45 | 0.60 |
| Commune ID: 2 ($C_{23}$) | -0.17 | -0.73 | 0.36 |
| Commune ID: 9 ($C_{24}$) | 0.21 | -0.33 | 0.74 |
| Commune ID: 8 ($C_{25}$) | 0.04 | -0.50 | 0.55 |
| Commune ID: 6 ($C_{26}$) | -0.17 | -0.74 | 0.36 |
| Commune ID: 26($C_{27}$) | -0.12 | -0.72 | 0.45 |
| Infants’ Age ($b_{2}$) | 0.04 | 0.01 | 0.08 |

PEI: Pneumococcal Exposure Index

Commune ID: Nha Trang is divided into 27 communes

**Table D:** The VIF values quantifying multicollinearity among the covariates of the model are shown here. The values are well below 10 (which, as a ‘rule of thumb’, is when multicollinearity becomes influential).

| **Covariate** | **VIF** |
| --- | --- |
| Mean PEI value | 1.05 |
| Infant Age | 1.03 |
| Commune | 1.08 |

PEI: Pneumococcal Exposure Index

VIF: Variance Inflation Factor

**Figure A:** The Directed Acyclic Graph of the factors involved in the spread of pneumococcus to infants in Nha Trang is shown here


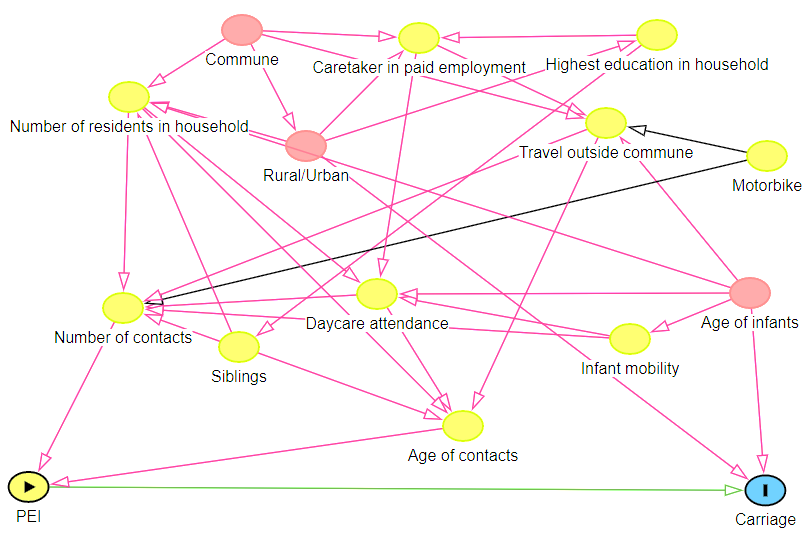


PEI: Pneumococcal Exposure Index

**Figure B:** Distribution of 10000 Markov Chain Monte Carlo (MCMC) samples of the Pneumococcal Exposure Index (PEI) for a randomly selected individual (Individual A), shown through a violin plot and boxplot nested within.


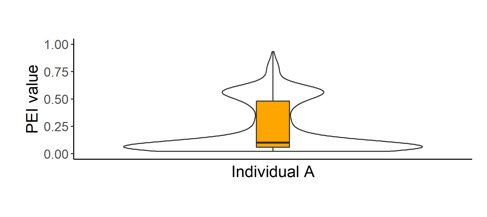


**Figure C:** Distribution of PEI values across different age groups for pneumococcal carriers and non-carriers.


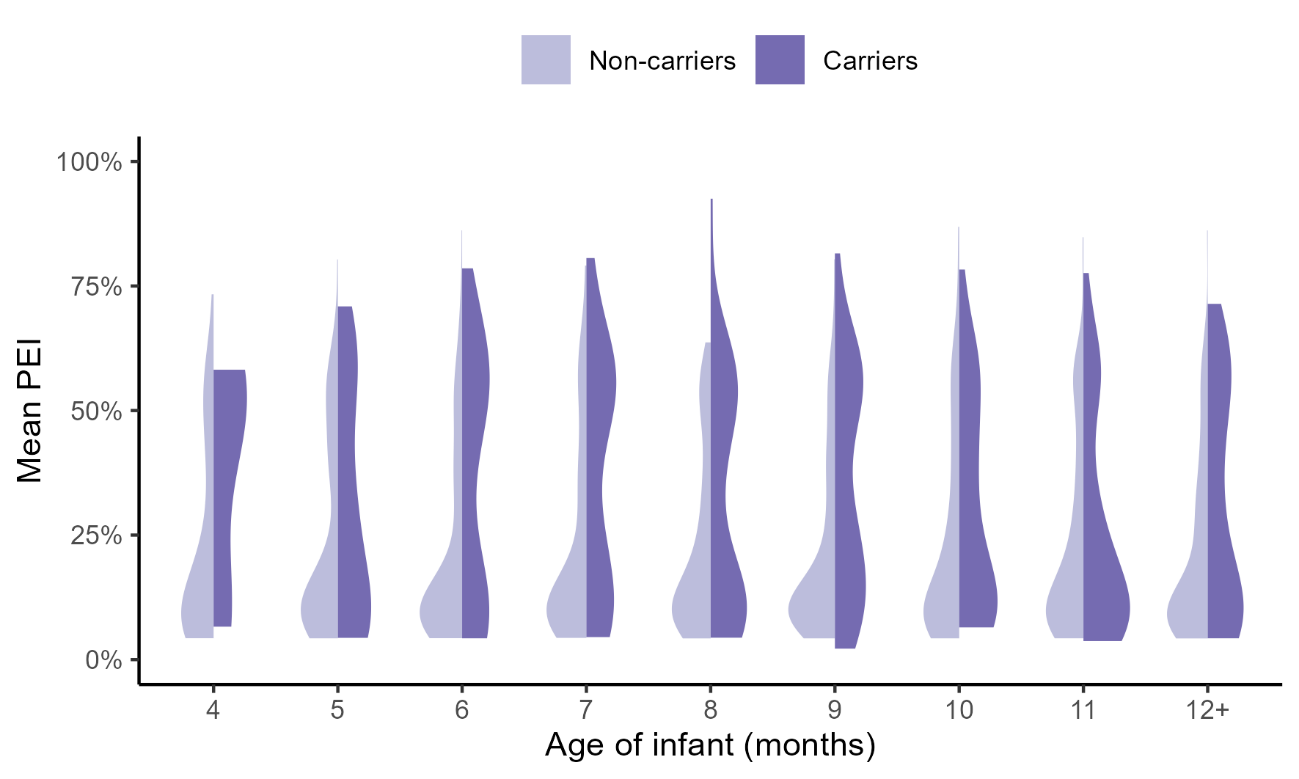

Supplement: S4 File — (DOCX) [file pmed.1004016.s005.docx]
