# Supplementary material for: Association of pneumococcal carriage in infants with the risk of carriage among their contacts in Nha Trang, Vietnam: A nested cross-sectional survey
Source: PLoS Med. 2022 May 31;19(5):e1004016. doi: 10.1371/journal.pmed.1004016 (PMC9197035; doi:10.1371/journal.pmed.1004016)
Supplement: S2 File — (PDF) [file pmed.1004016.s003.pdf]

Mã số: ___________________________

STT người tiếp xúc Contact Number	Người này là nam hay nữ, hay là một nhóm người? Chỉ chọn một cột. Is this person male or female, or a group of people? Just choose one option.			Tuổi (hoặc nhóm tuổi) What is the age or estimated age range of the person (or group)	Người này có phải thành viên trong gia đình bạn không? Is this person a member of your household		Địa điểm xảy ra cuộc tiếp xúc? (đánh dấu vào ô phù hợp) Where did your baby have contact? (tick one that apply)						Tổng thời gian tiếp xúc với trẻ Total time spent with the baby			Địa điểm tiếp xúc trong cùng xã/phường? Contact in the same commune?		Khoảng cách từ địa điểm tiếp xúc tới nhà (tính theo km)? Approximate distance from home (at contact)?	Trẻ có thường tiếp xúc với người này hoặc nhóm người này không? How often would your baby usually have contact with this person?				
	Nam Male	Nữ Female	Nhóm (ghi số lượng người) Group (give size)		Có Yes	Không No	Nhà Home	Nhà trẻ hoặc nơi chăm sóc trẻ Nursery or childcare	Phương tiện giao thông Transport	Nơi làm việc Work	Nơi vui chơi (khu mua sắm, khu bơi lội, công viên) Leisure (Shopping, Swimming, park etc.)	Khác (đi thăm ông bà, họp nhóm bà mẹ/trẻ em, v.v) Other (GP visits, mother / baby group etc)	≤5 phút 5≤mins	6 phút – 1 giờ 6 mins – 1 hours	>1 giờ >1 hour	Có Yes	Không No		Hàng ngày hoặc gần như hàng ngày Daily or almost daily	Một hoặc hai lần mỗi tuần Once or twice a week	Một hoặc hai lần mỗi tháng Once or twice a month	Ít hơn một lần mỗi tháng Less than once per month	Chưa gặp bao giờ Never met before
1																							
2																							
3																							
4																							
5																							
6																							
7																							
8																							
9																							
10																							
11																							
12																							
13																							
