# Supplementary material for: Association of pneumococcal carriage in infants with the risk of carriage among their contacts in Nha Trang, Vietnam: A nested cross-sectional survey
Source: PLoS Med. 2022 May 31;19(5):e1004016. doi: 10.1371/journal.pmed.1004016 (PMC9197035; doi:10.1371/journal.pmed.1004016)
Supplement: Attachment — Submitted filename: Letter to Editors and Reviewers_Submission.pdf [file pmed.1004016.s006.pdf]

Dear Editors and Reviewers,

Thank you for reviewing our manuscript describing the infant contact study carried out in Nha Trang, Vietnam. We very much appreciate the feedback and suggestions and have revised the manuscript accordingly.

Please find below a point-by-point response to each comment, including how we have modified the manuscript.

Finally, I'd just like to point out that we have added our collaborator (Catherine Satzke) to the list of authors. If this requires all authors to confirm that we are indeed happy with this change, please do let us know.

Thank you for your time and we look forward to hearing your response.

Yours sincerely,
George Qian, on behalf of all authors

Response to the Editors

Thank you for these comments. We have now made the appropriate changes; please see our responses here in this letter and in the manuscript.

1. Title: Please revise your title according to PLOS Medicine's style. Your title must be nondeclarative and not a question. It should begin with main concept if possible. "Effect of" should be used only if causality can be inferred, i.e., for an RCT. Please place the study design ("A randomized controlled trial," "A retrospective study," "A modelling study," etc.) in the subtitle (ie, after a colon).

We have revised the title of the manuscript to:
'Association of pneumococcal carriage in Vietnamese infants with the risk of carriage among their contacts: a nested cross-sectional survey'

2. Throughout: Please include line numbers with the revised version.

We have now added line numbers in the revised manuscript.

3. Abstract: Methods and Findings: Please explicitly mention the years during which the study took place and main outcome measures. Please specify the ages of the infants.

This information has now been added to the *Methods and Findings* section of the abstract: "*In October 2018, we conducted a nested cross-sectional contact and pneumococcal carriage survey in randomly selected 4 to 11 months old infants across all 27 communes of Nha Trang, Vietnam*"

4. Abstract: Methods and Findings: In the last sentence of the Abstract Methods and Findings section, please describe the main limitation(s) of the study's methodology.

This has now been added to the *Methods and Findings* section of the abstract: "*The main limitations of this study are that exposure risk was assessed indirectly by the age dependent propensity for carriage of a contact and not by assessing carriage of such contacts*"

5. Abstract: Conclusions: Please address the study implications without overreaching what can be concluded from the data; beginning with the phrase "In this study, we observed ..." may be useful.

We have now modified this part in the *Conclusions* section of the abstract: "*In this study, we observed cross-sectional contact and infection studies can help identify pneumococcal transmission routes and that preschool age children in Nha Trang may be the largest reservoir for pneumococcal transmission to infants.*"

6. Author Summary: At this stage, we ask that you include a short, non-technical Author Summary of your research to make findings accessible to a wide audience that includes both scientists and non-scientists. The Author Summary should immediately follow the Abstract in your revised manuscript. This text is subject to editorial change and should be distinct from the scientific abstract. Please see our author guidelines for more information: https://journals.plos.org/plosmedicine/s/revising-your-manuscript#loc-author-summary

Thank you. We have now added a non-technical Author Summary that adheres to the guidelines set out by PLOS Medicine. This appears just after the Abstract, as required.

7. Methods: "The study was conducted in Nha Trang, a coastal city in south-central Vietnam with a total population of just over 426,958 and under-five population of 28,495 in the 2018 census (personal communication with Khanh Hoa Health Service)."

The information may be cited in the text as a personal communication with the author if the author provides written permission to be named. Please provide the name of the individual, the affiliation, and date of communication. The individual named must provide written permission to be named. Alternatively, please provide a different appropriate reference.

We have provided the name of the individual, the affiliation, and date of communication as "(personal communication with Dr. Minh Xuan Bui, Khanh Hoa Health Service, October 2, 2021)" and attached written permission.

8. Methods: "In October 2016 a cluster randomised controlled trial was initiated to evaluate alternative pneumococcal conjugate vaccine (PCV) dosing schedules [13]." It would be helpful to describe relevant details of the trial, including recruitment and enrolment of participants. The Methods indicate that 24 of 27 communes were allocated to receive PCV vaccination. Please comment on how this would be expected to impact infant carriage and the PEI estimates, for example.

Details of the trial including vaccination schedules and how to sample participants are now described in the text (see reply to point #9 below). In brief, pneumococcal vaccination does not substantially change pneumococcal carriage prevalence due to serotype replacement. As our estimates for PEI are based on all serotype carriage this should not have been affected.

9. Methods: "In October 2018, two years after the start of the trial, an infant contact survey was nested into the cross-sectional carriage study." Please provide some information on the PCV vaccine schedule and coverage at the time when the survey was carried out. Please define how the infant population was sampled for the carriage study/contact survey. Please report the response rates, clarify the dates when the contact survey was conducted.

In the methods, we provided the PCV schedules as "two priming doses and one booster dose (2p+1), 3p+0, 1p+1, or 0p+1", the PCV coverage as "Completion rate of the routine vaccination in community was in 81.2%, 88.5%, 97.4%, and 87.0%, in 2p+1, 1p+1, 0p+1, and 3p+0 arms, respectively, using 18-months population as denominator for 2p+1, 1p+1, and 0p+1 arms and 12-months population for 3p+0 arm, in 2018 when an infant contact survey was conducted", and how we sampled the participants as "randomly selected from an administrative population list". We also added "Due to serotype replacement the trial did not affect overall carriage rates in the Nha Trang population". In the results, we described the response rate as "(response rate was 99%)" and the dates when the contact survey was conducted as "The contact study was conducted between 19th October and 7th November 2018". In addition to the actual dates, we included a little more detailed schedule of obtaining consent, contact information, and nasopharyngeal swab as "Consent to participate in the contact study and the nasopharyngeal carriage study was sought

from the carers by home visit. The date of keeping a contact diary was assigned and field workers conducted a second visit one or two days after to record the contact information based on the diary."

10. Methods: "In an adjusted analysis we included the age group (4-7 and 8-11 months of age)" Please explain the rationale for the categorization of ages into these groups.

The categories were selected as these were the first four months and the second four months of the target age range, in order to divide them into younger and older groups. This explanation has been added to the text (please see Line 168).

11. Methods: "Microbiological culture was subsequently attempted for lytA positive (cycle threshold (Ct) value < 35) or equivocal (Ct value 35-40) samples. Pneumococcal carriage was defined as samples that were both lytA positive/equivocal and culture positive, and subsequently confirmed by microarray [15] ." Please briefly describe the methods for qPCR detection, including primers, as well as for microbiological culture and microarray confirmation.

We have now expanded this description to the following (line 137 onwards):

Participants were also given an appointment with the commune health centre during a nasopharyngeal swab from the infant was obtained by a study nurse. Sample collection, handling and storage were performed according to World Health Organization guidelines [14]. At Pasteur Institute of Nha Trang, DNA extractions were performed using the QIAamp DNA Mini Kit (Qiagen), as previously described [15] and real-time quantitative PCR (qPCR) was performed using Forward primer: 5'-ACGCAATCTAGCAGATGAAGCA-3', Reverse primer: 5'-TCGTGCGTTTTAATTCCAGCT-3', and lytA Probe: 5' FAM - TGCCGAAAACGCTTGATACAGGGAG- 3' BHQ1 to detect the autolysin-encoding gene (lytA) of Streptococcus pneumoniae [16]. Microbiological culture was subsequently attempted for lytA positive (cycle threshold (Ct) value < 35) or equivocal (Ct value 35-40) samples. Samples containing alpha-haemolytic growth were tested by microarray (Senti-SPv1.5, BUGS Bioscience) at the Murdoch Children's Research Institute to determine serotype [17].

12. Methods: "We used negative binomial regression to estimate crude rate ratios of the mean number of contacts of participants in different population subgroups." Please define the population subgroups.

By ‘subgroups’, we were referring to the characteristics of the participants, such as male or female, with or without sibling(s), walking status, whether the infants attended daycare, etc. and all are defined in Table 1.
We have changed the word ‘subgroup(s)’ to ‘in each characteristic of the participants’ to avoid confusion, since ‘subgroups’ may well imply that our study included subgroup analysis. The phrase “defined in Table 1” has been added to the text. “Age at the time of enrolment” and “Transport available for immediate travel” were added to Table 1 for clearer definition of characteristics.

13. Methods: “To estimate age dependent probabilities for pneumococcal carriage in the study setting, we performed Bayesian Penalised B-Spline Regression on logit-transformed carriage probability, obtained from previously reported low-granularity age-stratified pneumococcal carriage across age groups from Nha Trang [16]. We compared our estimates against carriage prevalence observed in young children in the trial. The PEI estimate is based on the contact patterns recorded for a single day but were interpreted as a proxy for the general contact behaviour of the respective infant.”
Please provide additional description of the low-granularity age-stratified carriage data from reference 16 (including years when those data were obtained), as well as more description of how age dependent probabilities obtained were compared to the carriage prevalence observed in young children in the trial (including a reference for those data).

Thank you for pointing out that we haven’t given details of the data from the reference. These are now included in the text and we have also added that we use these data to compare against the credible intervals from our spline interpolation: “*These data were collected in October 2006 in a cross-sectional household survey. The age groups stratifications were: yearly until 5 years old and then 6-10y, 11-17y, 18-30y, 31-49y and over 50y old*.” And “*We visually validated our estimates against carriage prevalence observed in young children in the trial*”

14. Methods: Prospective analysis plan: Did your study have a prospective protocol or analysis plan? Please state this (either way) early in the Methods section.
a) If a prospective analysis plan (from your funding proposal, IRB or other ethics committee submission, study protocol, or other planning document written before analyzing the data) was used in designing the study, please include the relevant prospectively written document with your revised manuscript as a Supporting Information file to be published alongside your study, and cite it in the Methods section. A legend for this file should be included at the end of your manuscript.
b) If no such document exists, please make sure that the Methods section transparently describes when analyses were planned, and when/why any data-driven changes to analyses took place.

c) In either case, changes in the analysis-- including those made in response to peer review comments-- should be identified as such in the Methods section of the paper, with rationale.

Our study was mostly planned as a simple contact study and thus did not have a prospective analysis plan. We have now included the following (line 164): "*After completion of the nested contact survey we examined the association of infant infection and their contact behaviour but, in an extension to previous studies, also accounted for the risk of infection in each contact*."

15. Methods: STROBE: Please report your study according to the relevant guideline, which can be found here: http://www.equator-network.org/. Please ensure that the study is reported according to the STROBE guideline, if that is most appropriate, and include the completed STROBE checklist as Supporting Information. Please add the following statement, or similar, to the Methods: "This study is reported as per the Strengthening the Reporting of Observational Studies in Epidemiology (STROBE) guideline (S1 Checklist)."
The STROBE guideline can be found here: http://www.equator-network.org/reporting-guidelines/strobe/
When completing the checklist, please use section and paragraph numbers, rather than page numbers.

The STROBE checklist was attached as SI File 3 and a relevant statement was added to the text.

Following STROBE guidelines, we added to the Results section (line 247): "*Due to the carer's inconvenience" to explain non-participation, and also included (line 248): "There were no missing data in the contact survey except that S. pneumoniae carriage status could not be determined in one infant due to technical reasons. This infant was excluded from analysis of pneumococcal carriage*" to explain how missing data were addressed.

16. Results: Please present numerators and denominators for percentages, at least in the Tables (not necessarily each time they're mentioned).

Table 2 and S3 now have percentages with numerators and denominators.

17. Results: For the results described, please report in the text the results of all statistical analyses, with both CIs and p values.

We have now reported effect sizes, CIs, and p-values in the results.

18. Results: "Modelled carriage prevalence in Nha Trang based on carriage observations from a previous study…" please provide a reference for the study.

Thank you for pointing out that the reference isn't clear here; this is, in fact, the study from the low-granularity age-stratified carriage data (please see point number 13). We have now included the reference for this.

19. Discussion: Please present and organize the Discussion as follows: a short, clear summary of the article's findings; what the study adds to existing research and where and why the results may differ from previous research; strengths and limitations of the study; implications and next steps for research, clinical practice, and/or public policy; one-paragraph conclusion.

We have organised our Discussion section as suggested.

20. Tables and Supporting Information Tables: Please report p values in addition to confidence intervals presented for all analyses. Please also present the unadjusted results for all analyses. In the legends, please note statistical tests used, and factors adjusted for in the analyses.

Relevant p-values were added to Table 1 and Table S3. Unadjusted incidence rate ratios and unadjusted odds ratios are included in Table 1 and Table S3, respectively. Statistical tests used are in the titles and factors adjusted for in the analyses are in footnotes of Table 1 and Table S3.

21. Table S4: Please define “DIC” and “PEI” in the legend.
These have now been defined in the caption

22. Table S5: Please define “PEI” and explain “Commune ID” in the legend.
These have now been defined in the caption

23. Table S6: Please define “PEI” and “VIF” in the legend.
These have now been defined in the caption

24. Figure S4: Please define “PEI” in the legend.
These have now been defined in the caption

25. Figure S5: Please define “MCMC” and “PEI” in the legend.
These have now been defined in the caption.

Comments from the reviewers:

Reviewer #1: In the proposed paper, the authors report the results of a large pneumococcus carriage and contact tracing study from Vietnam. They develop an individual-level 'potential exposure index' based on the age-structure of contacts.

The descriptions of the number, location and type of contacts is an important addition to the literature, as is the age-specific probabilities of carriage. I only have a few comments.

Thank you for reviewing our manuscript; we very much appreciate these suggestions and have made the appropriate changes. Please see our responses below.

The authors claim that on average pneumococcal carriers had a higher PEI than non-carriers - however, the uncertainty with these number is (very) high. I do not believe the authors can make this statement - instead they should do a statistical test to see if the PEI levels were different between the two groups.

We agree and have subsequently performed the Mann-Whitney U test on the 2 groups. This shows that there is indeed a significant difference between the PEI values of carriers and non-carriers. We have added that:

"*The Mann-Whitney U test was used to assess whether there was a significant difference in average PEI value in carriers and non-carriers.*" to the Methods section (see Line 189) and:
"…; *and, from the Mann-Whitney U test, there was a statistically significant difference between PEI values from the two groups*" to the Results section (see Line 303)

It would be useful to understand the distributions of PEIs - overall and within each age group. At a population level each age group is assumed to have a normally distributed PEI values - but it is unclear how appropriate this is.

Thank you: the uncertainty in the individual level estimates for PEI in this analysis was retained from the posterior estimates in the previous regression (namely, the cubic spline on age) by assuming a normal distribution for each year of age and drawing samples from this distribution when calculating the posterior samples of PEI. We assumed a normal distribution because, by visual inspection, the distribution of carriage rates across all ages appeared to follow a Gaussian - please see Figure S6. We have also added a reference to this figure in the text (line 222).

The authors state that individuals that almost certainly had an exposure (PE=1) had a high odds of being positive - however, we do not know how common that is and what approximately 1 means (>0.95?). I assume that this implies that carriage within an age group is randomly distributed and that e.g., in a classroom of children each will have the same probability of being positive and their risks will not be correlated between members of the class - this should be stated and discussed.

PEI=1 indicates at least one effective contact with a pneumococcal carrier. This was unclear in our text and we have modified it accordingly to (see Line 306):

When adjusting for age and locality we find that the probability of exposure to pneumococci on the surveyed day and the risk of pneumococcal carriage were highly correlated: for every 10 percentage points increase in PEI the odds of pneumococcal carriage increased by 22% (95% CI: 15 - 29), i.e. the odds of pneumococcal carriage were 7.1 (95% CI: 4.1 - 12.3) times higher in infants who had at least one effective contact with a pneumococcal carrier (PEI = 1), compared to those who had not (SI Table 5).

I would move the logistic regression equation to the main text - it will make it clear what approach the authors have taken.

Thank you for this suggestion. We have done this now - please see the addition on line 199.

The authors assume a beta distribution for the PEI distributions - it wasn't clear how appropriate this assumption was - a histogram of the posterior with a beta distribution would help.

We assumed a beta distribution for PEI because PEI is bounded between 0 and 1, and each individual’s PEI distribution is right-skewed, as seen in SI Figure S5. While it cannot capture bimodality that we see in our PEI charts, we assume a beta distribution is nonetheless a satisfactory way to model PEI distributions. We have added this explanation to the text (page 9, line 218).

Subsequently, this individual-level uncertainty in PEI is further propagated by assuming a beta distribution for these PEI estimates, since PEI is bounded between 0 and 1, and each individual’s PEI distribution is right-skewed (see SI Figure S5).

It would be good to include a discussion of ways to improve PEI estimation - in particular in reducing uncertainty. For example, would looking at serotype/GPSC distribution help?

Thank you for this suggestion. We have added further material in the Discussion section on how to improve our PEI estimates, in particular (see Line 371 onwards):

- Carrying out a survey for more than one day
- Using a longitudinal survey with repeat sampling of infants and contacts, and sequencing of carriage isolates together with phylogenetic analysis.

Reviewer #2: In this study, the authors combine a high-quality dataset of pneumococcal carriage with detailed survey data to infer the contribution of different age groups to transmission of pneumococcus. The survey data are used to develop a novel index that quantifies an individual child's risk of exposure given their contacts

and the prevalence of pneumococcus in those contacts (by age). This is a clean, intuitive approach that aids in understanding the importance of different age groups for transmission. The authors also provide a well-documented Github repository, which should allow others to replicate the analyses in other settings.

Thank you for your comments and suggestions and we are pleased to receive such positive feedback. We have modified the manuscript to incorporate these comments (please see details below).

My only major comment is about the interpretation and directionality of the exposure index. For instance the authors "estimate that 2 to 6 year old children are key pneumococcal transmitters to infants, contributing 51% (95% CI: 39-63) of the total exposure, and that infection risk from other infants or older children and adults is likely low in this setting". Thinking about population-level effects, 51% of exposure is directly attributed to kids in that age group. But if the 2-6 year olds are also responsible for a large fraction of transmission to older children and adults, the effect would be even greater because the 2-6 year olds transmit to adults, who then transmit to infants. For example, in an extreme situation where all transmission in the population originated with 2-6 year olds, Is there a way to account for this type of dependency? If not, it should be clear that these estimates, which capture the direct contribution from the age group, represent a lower bound
for the total contribution of that age group.

Thank you for pointing out the indirect effects of transmission from certain age groups. We've now added a paragraph stating that we have only reported the direct effects and that, indeed, indirect effects from certain age groups may amplify their overall contribution to pneumococcal transmission. The paragraph reads (see Line 384):

It is also worth noting that our estimates for the relative proportions of exposure to infants represent only the direct contributions of each age group. Our study does not allow us to ascertain additional indirect effects of exposure that would arise when considering second generation (or beyond) exposure patterns.

The authors could also add some discussion about the implications of their findings for vaccination strategies. What is the potential policy response to knowing that 2-6 year olds are important for transmission? Catch-up campaigns? different dose timing?

Thank you for the comment. We do agree that this would benefit the paper and so have added a paragraph on vaccine strategies to the Discussion section (see Line 384 onwards).

Reviewer #3: This is an interesting paper combining pneumococcal carriage and social contact data to assess the risk of pneumococcal transmission as a function of social contacts among infants, building on previous studies in Uganda and Fiji.
The authors have used an elegant statistical approach to estimate the potential exposure to a pneumococcal carrier (and hence infection risk) based on knowledge about the social contacts of infants and the probability of carriage of their contacts. The method allows to further explore the type of social contacts that matter for pneumococcal transmission. The methods are clear and the paper is well written
I have few very comments on the paper:

Thank you for reviewing this manuscript. We are pleased to receive such feedback and give our response to the suggestions below.

1. In their calculation of the Potential Exposure Index, authors are using age-specific carriage probabilities. Have they looked at sex too, particularly among adult populations (caregivers, mothers). While the carriage prevalence in that setting is very low across all age groups, and the carriage difference between adult age groups is likely small too, it would be useful to explore

We agree that it would be useful to explore other covariates that may be associated with risk for pneumococcal carriage of a contact. However, we did not have the power to do this in this study. Further, we are not aware of substantial differences in carriage by sex that would a priori justify inclusion as a risk factor here.

2. It is a little unclear from the description whether there were variations among the study populations in vaccination schedule and/or coverage, given that this was nested within a trial, and whether carriage estimates differed between the different datasets used (pre-trial, trial). Please clarify

Information of vaccination coverage in each vaccination schedule arm in 2018 was added to the text as we replied to Editor’s comment #9 above.
In addition, carriage estimates between the pre-trial and trial datasets were found to be similar (falling within the 95% credible intervals of our spline interpolation).
