# Supplementary material for: Association of pneumococcal carriage in infants with the risk of carriage among their contacts in Nha Trang, Vietnam: A nested cross-sectional survey
Source: PLoS Med. 2022 May 31;19(5):e1004016. doi: 10.1371/journal.pmed.1004016 (PMC9197035; doi:10.1371/journal.pmed.1004016)
Supplement: Attachment — Submitted filename: Letter_To_Editors_May.pdf [file pmed.1004016.s007.pdf]

Dear Editors,

Thank you for again reviewing our manuscript describing the infant contact study carried out in Nha Trang, Vietnam. We very much appreciate the feedback and suggestions and have revised the manuscript accordingly.

Please find below a point-by-point response to each comment, including how we have modified the manuscript.

Thank you for your time and we look forward to hearing your response.

Yours sincerely,
George Qian, on behalf of all authors

Response to the Editors

Thank you for these comments. We have now made the appropriate changes; please see our responses here in this letter and in the manuscript.

1. Title: Please place the study design in the subtitle (ie, after a colon) and please update this in the manuscript submission system in addition to the main text. We suggest revising to: "Association of pneumococcal carriage in infants with the risk of carriage among their contacts in Nha Trang, Vietnam: A nested cross-sectional survey"

   Thank you: we have now changed the title to the one above

2. Abstract: Background: Please add a final sentence that clearly states the study question.

We have added the following sentence on lines 37 and 38: Hence, this study investigates pneumococcal transmission routes to infants in the coastal city of Nha Trang, Vietnam.

3. Abstract: Methods and Findings, Line 10: Please add the sentence punctuation: "In October 2018, we conducted a nested cross-sectional contact and pneumococcal

carriage survey in randomly selected 4 to 11 months old infants across all 27 communes of Nha Trang, Vietnam."

Thank you for noticing: we have now added the full stop at the end of this sentence (line 43).

4. Abstract: Methods and Findings: Line 16: Please report the actual numbers, in addition to percentages.

We have now added the numbers, along with the percentages on lines 49 and 50 e.g. *carriage prevalence was 22% (353 infants).*

5. Author summary: Please reformat the author summary, providing 2-3 single sentence bullet points under each of the following headings. Why Was This Study Done?; What Did the Researchers Do and Find?; What Do These Findings Mean? Please see https://journals.plos.org/plosmedicine/s/revising-your-manuscript#loc-author-summary for guidelines and examples.

We suggest reformatting as follows, or similar:

Why was this study done?
-There is little information in the current literature on pneumococcal transmission to infants. -Since individuals in this age group are at major risk of pneumococcal disease and at potentially reduced protection in reduced dose pneumococcal vaccination schedules, we carried out a study to identify their close contacts and likely routes of transmission.

What did the researchers do and find?
-We identified all 7428 physical contacts of 1583 infants in Nha Trang, a coastal city in Vietnam. -We found that there was a high correlation between infant infection and their probability of contact with at least one pneumococcal carrier on the surveyed day.
-Preschool-age children contributed around half of the total direct pneumococcal exposure risk to infants in Nha Trang. Around 80% of the exposure risk came from contact with household members.

What do these findings mean?
-Social contact surveys can help to identify possible transmission pathways.
- Infants in Nha Trang may be likely to receive substantial indirect protection against pneumococcal infection from vaccinated older siblings.

Thank you; we have reformatted the author summary to the suggested format above.

6. Methods: Line 93-94: Please also include a reference to the personal communication letter in the Supporting Information.

We have now added ‘see SI File 4’ on line 135.

7. Methods: Line 123, 128, and throughout: The terms gender and sex are not interchangeable (as discussed in http://www.who.int/gender/whatisgender/en/); please use the appropriate term.

Thank you for pointing out the inconsistency; we have now changed all terms to “sex” (line 169 in particular)

8. Methods: Line 134-135: Please specify if this is “written informed consent” for both contact and carriage studies.

We have now made this clear, and the sentence on lines 157-159 now reads: *During an initial home visit, written informed consent was obtained for both the infant contact and carriage studies from parents or guardians before any collection of data or specimen.*

9. Methods: Line 250: Please change this to “National Institute of Hygiene and Epidemiology in Hanoi, Vietnam”

We have now changed this; please see line 287

10. Methods: Line 163: Prospective analysis plan: Please explicitly state that the study had no prospective analysis plan. Please explicitly state when these analyses were planned, and when and why any data-driven changes to analyses took place, including those made in response to peer review comments.

The study had no prospective analyses plan. The analysis idea to combine contact and carriage data was conceived during a collaborative meeting that happened after the study was conducted but without being informed by the data.
Thus, we have added the following sentence to the ‘Statistical analyses’ subsection on line 205: The infant contact study is a cross sectional study and no prospective analysis is planned.

11. Results: Line 315-316: Please report the p value in the text for: “...from the Mann-Whitney U test, there was a statistically significant difference between PEI values from the two groups.”

Thank you; we have added the p-value now and the sentence now reads (see lines 352 and 353): *from the Mann-Whitney U test, there was a statistically significant difference between PEI values from the two groups ($p < 0.001$).*

12. Lines 418-430: Please remove the “Author contributions” and “Funding” sections from the manuscript text, and please make sure all information is completely and accurately entered into the relevant sections of the manuscript submission system.

Thank you; these sections have now been removed

13. Figures and Tables: Please be sure that each text, table, and figure has a descriptive title, and a legend describing the figure/table. We suggest numbering the figures/tables within the supporting information separately from the main text (begin with S1 Table, for example).

Thank you; please now find the captions to the main figures on lines 624-642; the descriptive titles for the tables remain as they were. The numbering of the supplementary information has been changed to the suggested format.

14. Table S3: Please report p values in addition to 95% CIs.

We have now added the p-values to Table S3; 95% CIs are included too

15. References: Please check each reference and please use the "Vancouver" style for reference formatting, and see our website for other reference guidelines https://journals.plos.org/plosmedicine/s/submission-guidelines#loc-references

Please check journal title abbreviations. For example, reference 3 should be formatted as PLoS Med.

Thank you; we have gone through each reference and made the appropriate changes.
